# Image dataset of pomegranate fruits (*Punica granatum*) for various machine vision applications

**DOI:** 10.1016/j.dib.2021.107249

**Published:** 2021-06-26

**Authors:** Arun Kumar R, Vijay S. Rajpurohit, Nilesh N. Gaikwad

**Affiliations:** a Tontadarya College of Engineering, Gadag, Affiliated to Visvesvaraya Technological University, Belagavi, India; b KLS Gogte Institute of Technology, Belagavi, Affiliated to Visvesvaraya Technological University, Belagavi, India; c ICAR-National Research Center on Pomegranate, Solapur, India

**Keywords:** Pomegranate, Image dataset, Pomegranate fruit database, Image database of pomegranates

## Abstract

Dataset - an essential aspect and the requirement for any of the machine learning project. Collection/creation of dataset in the agriculture domain is highly challenging task because the domain itself is uncertain. Main objective of the present paper is to create an image dataset of pomegranate fruits of different grades. Accordingly, we have considered ‘Ruby’ cultivar of pomegranate and sincerely constructed the dataset. Fruits belonging to three grades are considered. The images for each fruit are covered from all the three angles. The dataset created also contains the weights of the fruits. The dataset consists of 12 folders named after their effective quality grades. The usage of this dataset is already proved in the works carried out by the authors in their previous studies. This dataset is highly helpful for the data science engineer / machine learning programmer or machine learning expert working in the field of precision agriculture.

## Specifications Table

**Table:** 

Subject	Agricultural Sciences
Specific subject area	Image dataset of Pomegranate Fruits (*Punica granatum*) of different grades
Type of data	Table Image
How data were acquired	In the present work, data were acquired using two instruments viz. (1) Weighing Machine and (2) Image acquisition unit (Custom-built) with provision to place light source and cameras.
Data format	Raw
Parameters for data collection	The images are captured using Logitech C905 720p Webcam with 2MP sensor using the Logitech (R) Webcam software Version 2.50 under CFL (Compact Fluorescent Light) as the light source.
Description of data collection	The weighing machine is used to measure the weight of the fruit sample. Image acquisition unit is a custom-built metallic compartment in which object of interest (i.e., pomegranate fruit), light sources and cameras can be positioned within. The unit is designed in such a way that each of the fruit can be imaged in all the three angles. Further, the fruits are imaged, and their weighs are measured every alternate day for a duration of eight days, resulting into four qualities within each grade of the fruit.
Data source location	Institution: Private Farm Land City/Town/Region: Kaladgi, Bagalkot District, Karnataka state. Country: India Latitude and longitude (and GPS coordinates, if possible) for collected samples/data: 16°12′17.863″N 75°30′5.27″E, Altitude: 452 msl
Data accessibility	Repository name: kaggle Data identification number: 10.34740/kaggle/ds/551234 Direct URL to data: https://www.kaggle.com/kumararun37/pomegranate-fruit-dataset
Related research article	Arun Kumar R, Vijay S. Rajpurohit, and Kshitijarun Y. Bidari, Multi Class Grading and Quality Assessment of Pomegranate Fruits Based on Physical and Visual Parameters, International Journal of Fruit Science. 19 (2018) 372-396. https://doi.org/10.1080/15538362.2018.1552230

## Value of the Data

• The dataset is important as for as grading of pomegranate fruits is considered. Specifically, the image dataset along with weight as the physical parameter is scares. Hence the dataset is important for the purpose of automated quality grading during post-harvest processing of pomegranate fruits.
• The dataset built is made available to the public domain. Such a dataset is of great input for various researchers in building machine learning algorithms for quality grading of pomegranate fruits.
• The data may be used/reused by conducting experiments related to the quality grading of pomegranate fruits various machine learning algorithms, apart from the algorithms that authors have incorporated, as provided in the specifications table above. Moreover, the researchers who are involved in automated quality inspection of other fruits also may get benefited indirectly.
• Building the grade-wise image dataset of pomegranate fruits along with their weights is the uniqueness of the dataset. Moreover, each fruit is imaged in all the three views.

## 1 Data Description

The dataset consists of three grades and four qualities for each grade. Accordingly, there are twelve folders. Each folder is titled after its corresponding quality grade as outlined in Table 6. In each folder there are 90 images corresponding to the images of three views of 30 sample fruits. We have used the following syntax in naming each image:

<GN_QN_FSN_IVN>
**Legends:**
***GN***: Grade Number - Representing the grade value of the fruit.
***QN***: Quality Number - Representing the quality value of each grade.
***FSN***: Fruit Serial Number - Representing the serial number of fruit. Since 30 sample fruits are considered, the range of FSN is 1 to 30
***IVN***: Image View Number - Representing the view number or the angle number. Since each fruit is imaged in three angles, value of IVN range from 1 to 3.

Additionally, each folder consists of an excel sheet representing the weights of 30 sample fruits of each of the corresponding quality category.

## 2 Experimental Design, Materials and Methods

One of the hardest problems that every programmer faces in the development of machine vision applications / solutions is the availability of *right dataset*. Machine Learning depends heavily on data without which it is impossible to train any of the algorithms to recognize patterns. It is the most important aspect that makes algorithm training achievable. The accuracy of the training depends heavily on the quality of the dataset input [1]. Creating such a dataset is not always an easy stuff.

There are distinctive problems associated with the agricultural and horticultural industries viz. (1) High rise losses in post-harvest (2) Labor requirements (3) Subjectivity (4) Tediousness (5) Inconsistency etc. One of the main causes in the lowered product quality is the huge number of losses during post-harvest that can be found at variegated stages of marketing [2]. However, the studies have proved that all such problems associated with post-harvest losses can be effectively addressed by coalescing Digital Image Processing and Machine Learning techniques at variegated stages of post-harvest processes.

Post-harvest handling of fruits is vital in the horticulture domain as fruits are the important supplement to the human diet. Moreover, production of fruits in India holds an average of 31.3% share in the total production of horticulture crops in the last 5 years [3]. Pomegranate grabs the attention among all fruits as India is one of the biggest producers of pomegranates throughout the world and there is an absurd latent in exporting pomegranate fruits from India.

Grading is one of the important steps of the post-harvest management that is used to arrive at a reasonable pricing of pomegranates in both domestic and export markets. Continued boost in image processing and machine learning domains can provide effective tools and techniques in building systems that are capable of grading the pomegranate fruits provided a right dataset to the learning algorithms. Accordingly, goal of the present proposed work is a sincere effort in building a dataset of pomegranate fruits that aids in developing machine vision-based applications including Grading, Quality assessment and Sorting.

To the best of our knowledge, there is no public dataset available specifically for the gradation purpose of pomegranate fruits. Hence, there is a great need for building the dataset of pomegranate fruits.

There are various researchers working around the globe in fruit grading using machine vision in place. Present section outlines few of the research works consisting of pomegranate fruits. Authors in [8] classified the diseased pomegranate fruits and healthy ones using their own set of images. Quality of pomegranates was evaluated in [9] non-invasively by considering locally sourced Turkish pomegranates. Non-destructive pomegranate fruit grading and classification was carried out in [10] by using the cofilab dataset [8]. Identifying disease on pomegranate fruits using image processing was carried out in [7] using custom built images. Sunburn on pomegranate fruits was identified in [4] using custom built images. From the literature review, it is witnessed that the image datasets of pomegranate fruits are highly scarce. Hence there is a great need to build the dataset of pomegranate fruit images. Table 1 summarizes the previous works in connection with image processing of pomegranate fruits.

**Table 1:** Summary of works related to image processing of pomegranate fruits.

Sl. No.	Citation	Objective of the study	Dataset	Remarks
1	[4]	Detecting sunburn on pomegranate fruits	Custom built	20 fruit samples are used for detecting the sunburn
2	[7]	Identification of pomegranate fruit disease	Custom built	Images are used for the purpose of disease identification
3	[8]	Segregate diseased and healthy pomegranates	Custom built	Images are used for the purpose of segregating diseased and healthy fruits
4	[9]	Non-invasive quality assessment of Turkish pomegranates	Locally collected	15 pomegranate fruits are considered and stored in the controlled environment
5	[10]	Non-destructive fruit grading and classification of Wonderful pomegranates	cofilab pomegranate digital database [8]	Images are obtained from two angles

In the current work, ‘Ruby’ cultivar of pomegranate fruit is considered and accordingly the dataset is built. There are three grades considered for collecting the dataset. There are four persons involved in the grading process along with the corresponding author and all the authors have expressed their sincere gratitude in the acknowledgement section for the personnel involved in this process.

Table 2 briefs the description about the data collection.

**Table 2:** Brief description about the data collection.

Sl. No.	Particulars	Description
1	Fruit	Pomegranate
2	Cultivar	Ruby
3	Number of grades considered	3
4	Count of samples of each grade	30
5	Total count of samples	90
6	Geographical location	16°12′17.863″N 75°30′5.27″E Altitude: 452 msl
7	Atmospheric conditions during harvest of the fruits	Temperature: 26 °C Wind: 31 km/h Gust: 41 km/h Humidity: 69% Pressure: 1008 mb

Table 3 outlines the description associated with each of the three grades.

**Table 3:** Characterization of the grades.

Sl. No.	Grade	Range of weights (gm/fruit)	Remarks
1	G1	300 to 400	Smooth surface, Minor superficial defects that does not alter the quality and look
2	G2	200 to 300	Minor irregularities may be present such as scar, scrape, scratch, blemish etc. these irregularities will not affect the look and quality
3	G3	100 to 200	Minor irregularities may be present such as scar, scrape, scratch, blemish etc. these irregularities will not affect the look and quality

**Fig. 1:**
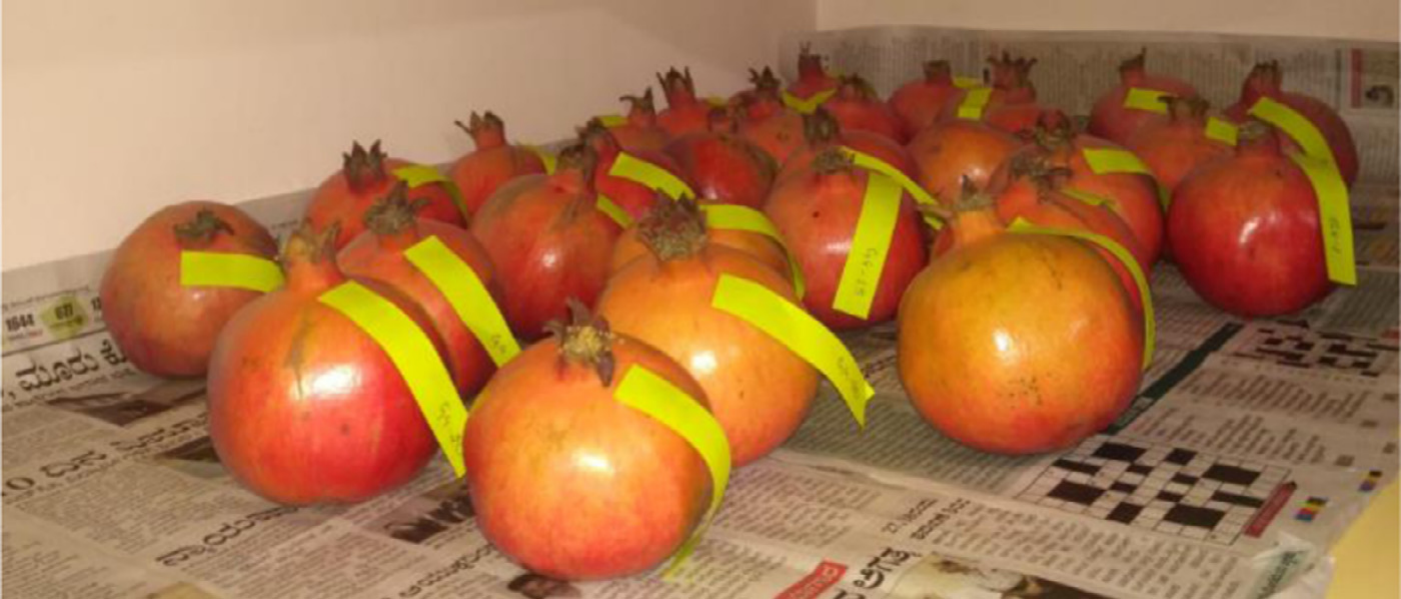
Tagging process of pomegranate fruit samples.

In the present work, pomegranate fruits are collected and are preserved for duration of eight days for the purpose of analysis. The storage conditions are as follows: Average Temperature: 22 °C, Wind: 20 km/h, Gust: 25 km/h, Humidity: 90%, Pressure: 1005 mb. The preserved fruit samples are imaged for every alternate day. Such an analysis resulted the creation of four qualities within each grade. Hence, a total of twelve classes of effective qualities got created. Since the fruit samples are preserved for some duration, tagging of each fruit is done so as to keep track of each fruit. Fig. 1 shows sample tagging of pomegranate fruits preserved for analysis. The designations for each class label are given in Table 4. Table 5 summarizes the description of the effective qualities in brief. Readers of this article are encouraged to refer the work carried out by the authors [6] for further details and applications of the qualities in each grade. The quality definitions are same for each of the three grades i.e., for example the fruits belonging to G1Q1 or G2Q1 or G3Q1 all bear the same visual characteristics and applications of Q1 except physical characteristics.

**Table 4:** Class labels of pomegranate quality grades.

Class	1	2	3	4	5	6	7	8	9	10	11	12
Effective Quality	G1- Q1	G1- Q2	G1- Q3	G1- Q4	G2- Q1	G2- Q2	G2- Q3	G2- Q4	G3- Q1	G3- Q2	G3- Q3	G3- Q4

**Table 5:** Description of the quality definitions.

Quality	Physical characteristics		Visual characteristics		
	Size (Diameter)	Weight	Outer surface	Color	Texture
Q1	Largest	Highest numerical value for Grade G1, Intermediate numerical value for G2 and Lowest numerical value for G3	Free from defects like scar, scratch, scrape, blemish and dents	Dark red or rose pink	Smooth and shiny surface
Q2	-NA-	Not less than 98% of the Q1 for G1, Not less than 98% of the Q1 for G2 and not less than 97% of the Q1 for G3	Free from defects like scar, scratch, scrape, blemish and dents	Dark red or rose pink	Smooth and shiny surface
Q3	-NA-	Not less than 98% of the Q2 for G1, Not less than 98% of the Q2 for G2 and not less than 97% of the Q2 for G3	Possibility of minor dents and blemishes	Reddish brown	Surface loses its luster
Q4	-NA-	Not less than 97% of the Q3 for G1, Not less than 96% of the Q3 for G2 and Not less than 95% of the Q3 for G3	Appearance of dents and blemishes	Dull Reddish brown	Rough surface

### 2.1 Weight measurement

Following Table 6 specifies the weighing machine used in the current work to measure the weight of each fruit sample.

**Table 6:** Specifications of the weighing machine.

Sl. No.	Particulars	Details
1	Make	Electronic Kitchen Scale
2	Model	SF-400
3	Sensor	Strain gauge sensor
4	Capacity	1000g*1g
5	Display	LCD

### 2.2 Image acquisition unit

**Fig. 2:**
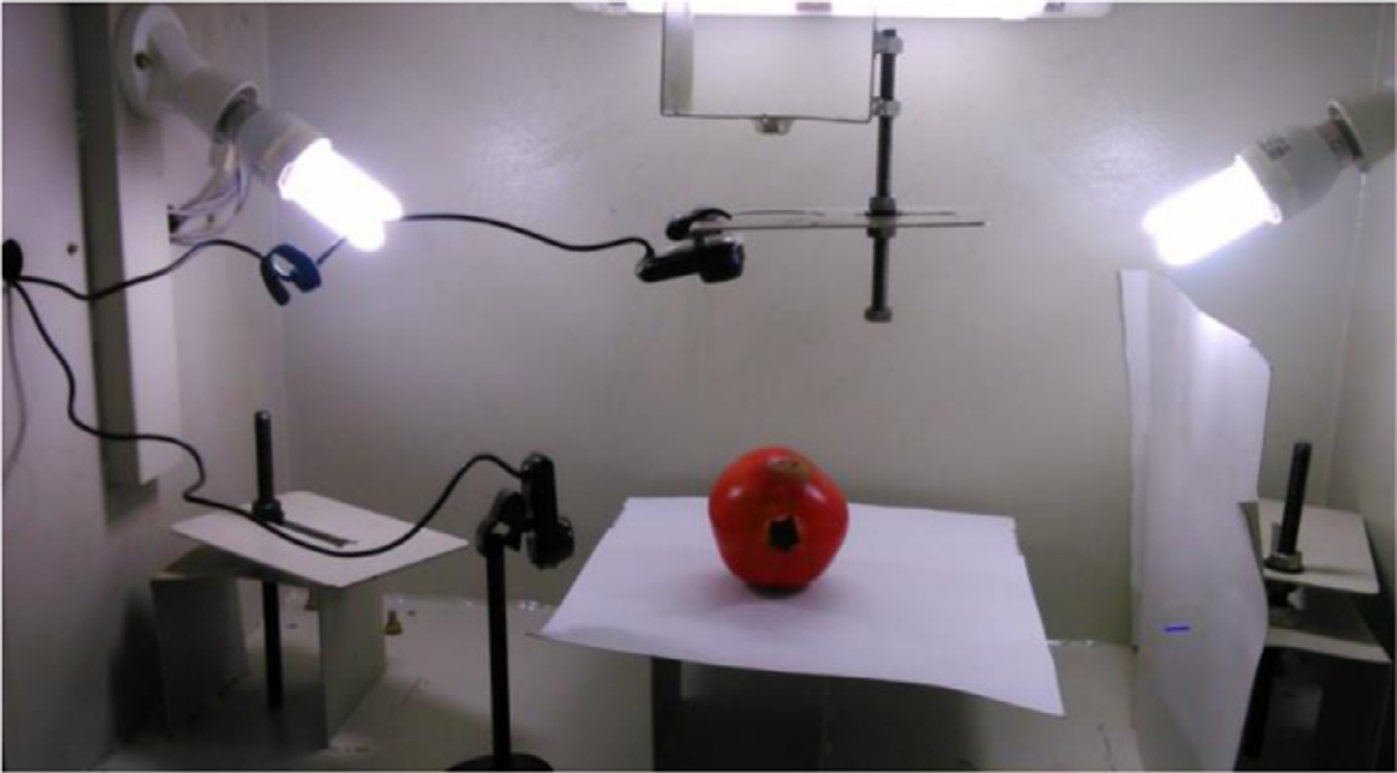
Image acquisition unit.

Images are formed by a blend of the source of illumination and the energy reflection by the objects in the scene [5]. In the present work, the compact fluorescent light is the illumination source and objects are the pomegranate fruits. An image acquisition compartment is custom built for the purpose of image acquisition, there by mimicking the industrial packing lines. The compartment is a metallic one in which object of interest (i.e., pomegranate fruit), light sources and cameras can be positioned within. This image acquisition unit is represented in Fig. 2. In order to cover the entire fruit surface area, each of the pomegranate fruits is imaged from all the three angles / views. Following Table 7 gives the specifications of the image acquisition.

**Table 7:** Specifications of the image acquisition.

S1. No.	Particulars	Details
1	Light origin	(i) CFL (Compact Fluorescent Light) (ii) Voltage: 240 V (iii) Frequency: 50 Hz (iv) Current: 65 mA (v) Power factor: 0.85, 765 Lumen
2	Camera	(i) Make and Model: Logitech C905 720p Webcam (ii) Sensor: 2MP (iii) Focal length: 3.7 mm (iv) Lens aperture f/2.0 (v) Focus adjustment: automatic, auto-exposure mode
3	Acquisition software	Logitech (R) Webcam software Version 2.50
4	Resolution of image	1600 × 1200 96 dpi (3.779528 pixel/mm)
5	Image format	Jpeg

Finally, the time span of the data collection is summarized in Table 8.

**Table 8:** Time span table.

Sl. No.	Date (DD/MM/YYYY)	Activity	Remarks
	24/06/2018	Fruit harvest and grading	-Nil-
1	25/06/2018	Image acquisition and weight measurement	Resulted into quality: Q1
2	27/06/2018		Resulted into quality: Q2
3	30/06/2018		Resulted into quality: Q3
4	03/07/2018		Resulted into quality: Q4

## Ethics Statement

There is no funding for the present effort. There is no conflict of interest. The data is available in public domain.

## CRediT Author Statement

**Arun Kumar R:** Methodology, Software, Validation, Formal analysis, Writing - Original Draft, Visualization, Funding acquisition; **Vijay S. Rajpurohit:** Conceptualization, Writing - Review & Editing, Supervision, Project administration; **Nilesh N. Gaikwad:** Resources, Writing - Review & Editing.

## Declaration of Competing Interest

The authors declare that they have no known competing financial interests or personal relationships which have or could be perceived to have influenced the work reported in this article.

## References

[1] Mureşan H., Oltean M (2018). Fruit recognition from images using deep learning. Acta Universitatis Sapientiae, Informatica.

[2] Murthy D.S., Gajanana T.M., Sudha M., Dakshinamoorthy V. (2009). Marketing and post-harvest losses in fruits: its implications on availability and economy. Indian J. Agric. Econ..

[3] Ministry of Agriculture & Farmers’ Welfare Department of Agriculture, Cooperation & Farmers’ Welfare Horticulture Statistics Division (2018). Horticultural Statistics at a Glance.

[4] Rezaei P., Hemmat A., Shahpari N. (2018). Detecting sunburn in pomegranates using machine vision. Electrical Engineering (ICEE), Iranian Conference on.

[5] Gonzalez Rafael C., Woods Richard E. (2009). Digital Image Processing.

[6] Kumar R A., Rajpurohit V.S., Bidari K.Y. (2019). Multi class grading and quality assessment of pomegranate fruits based on physical and visual parameters. Int. J. Fruit Sci..

[7] Lamani S.B. (2018). Pomegranate fruits disease classification with K means clustering. Int. J. Res. Trends Innov..

[8] Image Database: Half Cut Pomegranates. http://www.cofilab.com/portfolio/image-database-pomegranate/ (accessed 14 December 2019). Cancer Research UK, Cancer statistics reports for the UK.

[9] Czieczor L., Bentkamp C., Damerow L., Blanke M. (2018). Non-invasive determination of the quality of pomegranate fruit. Postharvest Biol. Technol..

[10] Gurubelli Y., Ramanathan M., Ponnusamy P. (2019). Fractional fuzzy 2DLDA approach for pomegranate fruit grade classification. Comput. Electron. Agric..

